# The knotted appendix: an unusual cause of bowel obstruction: a case report

**DOI:** 10.1093/jscr/rjaf520

**Published:** 2025-07-14

**Authors:** Suleiman Ayalew Belay, Muluken Assefa Zemariam, Michael A Negussie, Mesenbet Tsegaye Ferede, Yirgedu Getu Tadele, Yonas Admaw Tiruneh, Hailemariam Yohannes Asefa

**Affiliations:** School of Medicine, College of Medicine and Health Sciences, University of Gondar, Maraki Street, Gondar City, Central Gondar Zone, PO Box 196 Gondar, Ethiopia; Department of Surgery, College of Medicine and Health Sciences, University of Gondar, Maraki Street, Gondar City, Central Gondar Zone, PO Box 196 Gondar, Ethiopia; School of Medicine, College of Health Sciences, Addis Ababa University, Tikur Anbessa Specialized Hospital, Churchill Avenue, Lideta Sub-City, PO Box 5657 Addis Ababa, Ethiopia; Department of Surgery, College of Medicine and Health Sciences, University of Gondar, Maraki Street, Gondar City, Central Gondar Zone, PO Box 196 Gondar, Ethiopia; Department of Surgery, College of Medicine and Health Sciences, University of Gondar, Maraki Street, Gondar City, Central Gondar Zone, PO Box 196 Gondar, Ethiopia; Department of Surgery, College of Medicine and Health Sciences, University of Gondar, Maraki Street, Gondar City, Central Gondar Zone, PO Box 196 Gondar, Ethiopia; School of Medicine, College of Medicine and Health Sciences, University of Gondar, Maraki Street, Gondar City, Central Gondar Zone, PO Box 196 Gondar, Ethiopia

**Keywords:** small bowel obstruction, appendicular knot, strangulated, case report

## Abstract

Small bowel obstruction (SBO) is a common surgical emergency, but appendico-ileal knotting is an exceptionally rare cause. This condition involves the appendix forming a constricting loop around the ileum, potentially leading to closed-loop obstruction and bowel ischemia. We report the case of a 57-year-old male from Ethiopia who presented with classical features of SBO, including abdominal pain, bilious vomiting, and failure to pass stool and flatus. Imaging was inconclusive, and an exploratory laparotomy revealed appendico-ileal knotting involving the terminal ileum. The bowel was viable, and a simple appendectomy was performed. The patient had an uneventful recovery and was discharged on postoperative Day 5. Histopathology confirmed acute appendicitis with mucocele. This case underscores the diagnostic challenge of this rare condition and emphasizes the need for early surgical intervention to avoid complications.

## Introduction

Small bowel obstruction (SBO) is a prevalent surgical emergency, accounting for ~75% of all mechanical intestinal obstructions [1]. The appendicular knot, also known as appendicular band syndrome or appendicular tie syndrome, is an exceptionally rare surgical condition with only a few reported cases [2]. Hotchkiss *et al.* first described this condition in 1901 [3], and since then, only a handful of cases have been documented. This condition typically manifests as intestinal obstruction, where the ileum becomes entrapped by the appendicular knot, leading to a closed-loop obstruction. If not promptly addressed, it may result in strangulation and small bowel gangrene [4]. Preoperative diagnosis is challenging and usually made during laparotomy. We report a case of a 57-year-old male patient who presented with SBO secondary to appendico-ileal knotting, diagnosed intraoperatively.

This case report adds meaningful knowledge to the scarce existing literature and offers further understanding of this uncommon cause of SBO, especially within a resource-constrained setting.

## Case presentation

A 57-year-old male from northern Ethiopia presented to the emergency department with a week-long history of crampy periumbilical abdominal pain. This was associated with vomiting of ingested material, which later turned bilious. Additionally, he experienced a three-day history of failure to pass feces and flatus. He had no prior abdominal surgeries or chronic medical conditions.

**Figure 1:**
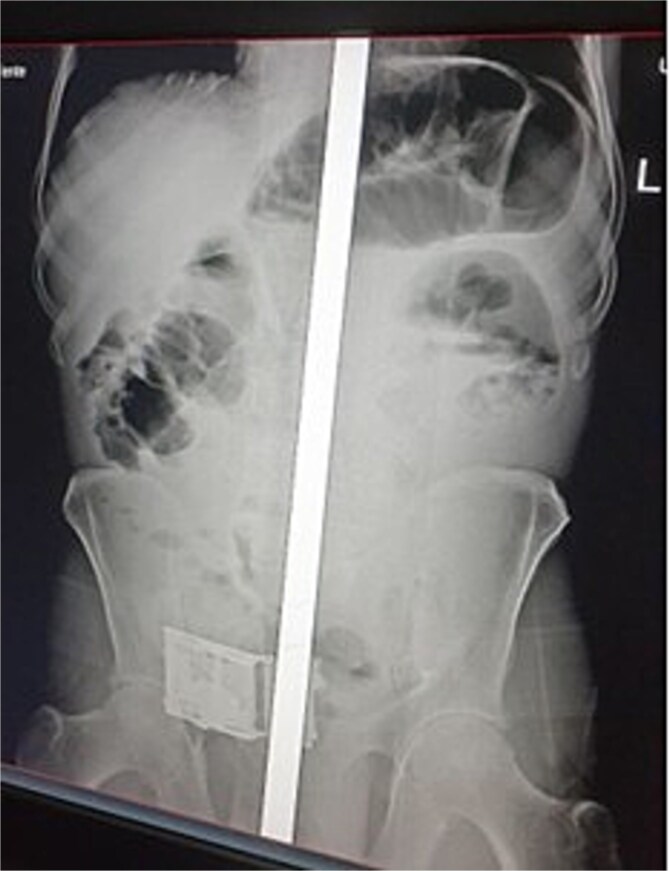
Abdominal radiograph showing multiple distended loops of small bowel.

On examination, his vital signs were within normal limits. His abdomen was slightly distended with visible dilated bowel loops but no tenderness. A rectal examination revealed a collapsed, empty rectum. A plain abdominal X-ray showed visible dilated bowel loops (Fig. 1). Blood tests revealed normal complete blood count and organ function tests. An abdominal CT scan was not performed due to the temporary unavailability of the CT scanner.

**Figure 2:**
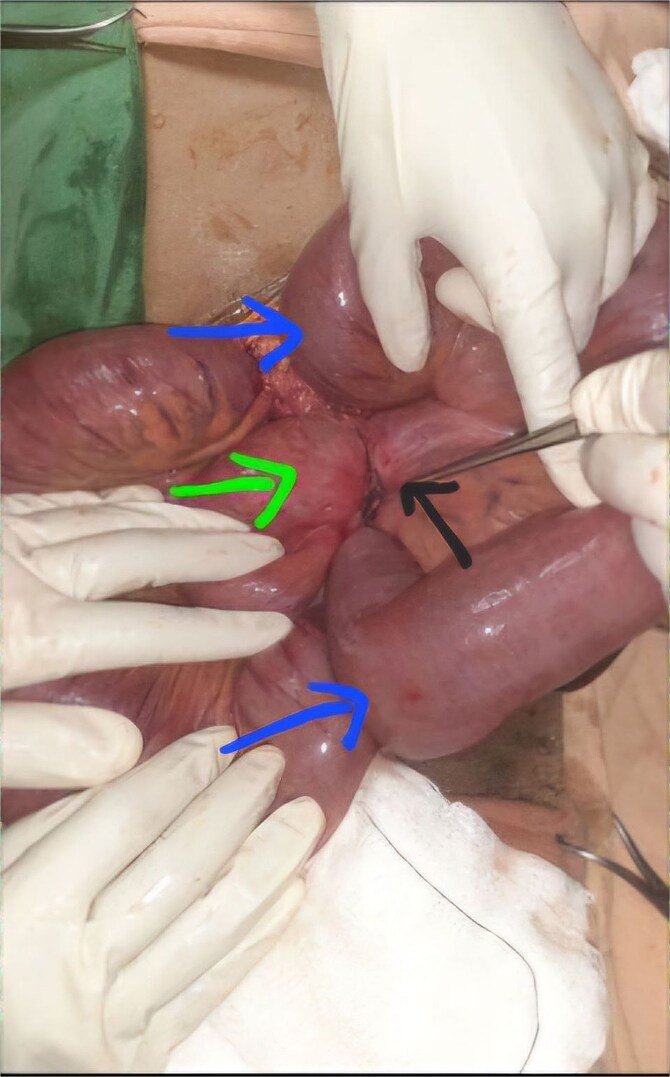
The appendix (black arrow) encircling the loops of terminal ileum (green arrow) with dilatation of proximal bowel loops (blue arrows).

**Figure 3:**
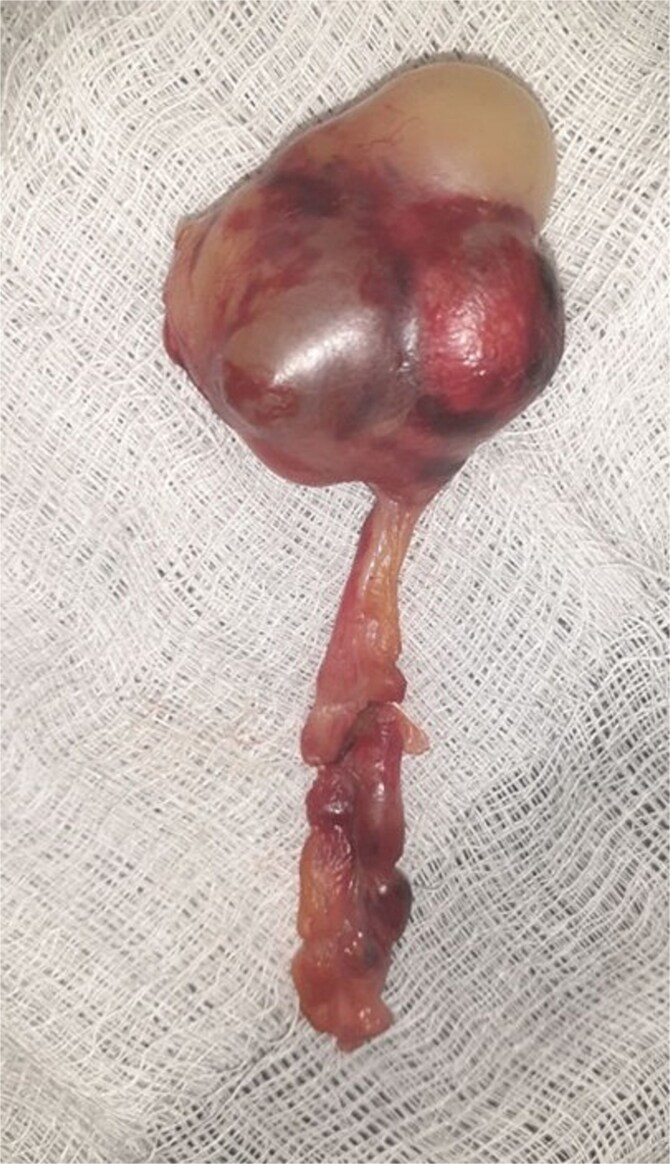
Showing the appendix with multiple cystic mass.

With a preoperative diagnosis of mechanical SBO of unknown etiology, the patient underwent emergency exploratory laparotomy. A midline laparotomy was performed, revealing 100 ml of reactive fluid in the peritoneal cavity. The appendix was found constricting the terminal ileum, involving the distal 10 cm of the ileum (Fig. 2). The appendix had a 2 × 3 cm multicystic mass at the tip, 4 cm from the base (Fig. 3), but there was no mesenteric lymphadenopathy. The reactive fluid was aspirated. As the bowel was viable, a simple appendectomy was performed, and the abdomen was closed in layers. The patient was then transferred to the Post Anesthesia Care Unit with stable vital signs.

Postoperatively, the patient had an uneventful recovery. Oral feeding was initiated after ~48 hours with evidence of bowel movement. He was subsequently discharged on the 5th postoperative day. Histopathology of the excised appendix revealed acute appendicitis with mucocele.

## Discussion

In 1909, Hawkes [5] categorized the causes of SBO due to acute appendicitis into mechanical and septic appendicitis, or a combination of both. The appendix is a mobile organ with variable positioning, and during appendicitis, it tends to adhere to surrounding structures, resulting in mechanical SBO. An increased length of the appendix seems to facilitate this phenomenon [6].

In 2009, Bhandari *et al.* [7] classified intestinal obstruction due to appendicitis into four types: adynamic, mechanical, strangulation, and mesenteric ischemia. Among the mechanical causes, the majority are due to appendicular abscess formation, which compresses the small bowel loops, and postoperative adhesions occurring years after treatment [7]. There are two primary scenarios where the appendix may cause mechanical obstruction: the appendicular tip attaching to the intestinal serosa, causing obstruction by direct compression or torsion, and the appendicular tip attaching to the mesentery surrounding an ileal loop, causing lumen compression [6], as observed in our case.

Preoperative diagnosis of appendico-ileal knotting as a secondary cause of SBO is challenging. Appendico-ileal knotting lacks specific features distinguishing it from other SBO causes. Misdiagnosis may occur due to the rarity of cases and limited clinical experience. Though a radiologist's expertise is crucial, the scarcity of imaging modalities like abdominal CT scans limits diagnostic capacity, particularly in resource-limited settings [8]. Typically, it is diagnosed intraoperatively. Management options are determined by intraoperative findings.

The management of appendico-ileal knotting depends on the involved bowel parts and the level of strangulation. Surgical treatment ranges from appendectomy to resection of the gangrenous bowel. Intraoperatively, if the bowels are viable, untying the knot and performing an appendectomy may suffice, as in our case. However, if the bowels are ischemic, non-viable, or gangrenous, bowel resection and anastomosis are required [9]. Weldegiorgis *et al.* [10] recently reported a comparable case involving gangrenous bowel caused by appendico-ileal knotting. The condition was managed through an initial procedure involving ileostomy, appendectomy, and hemicolectomy, followed by a subsequent ileostomy reversal with anastomosis.

## Conclusion

Appendico-ileal knotting is a very rare cause of intestinal obstruction. High index of suspicious is very important in order to make a diagnosis since delay in diagnosis may carry high risk of morbidity and mortality. Early diagnosis and treatment have a good outcome.
